# Interpretation of arterial blood gas

**DOI:** 10.4103/0972-5229.68215

**Published:** 2010

**Authors:** Pramod Sood, Gunchan Paul, Sandeep Puri

**Affiliations:** **From:** Critical Care Division, Dayanand Medical College and Hospital, Ludhiana, Punjab, India; 1 Department of Medicine, Dayanand Medical College and Hospital, Ludhiana, Punjab, India

**Keywords:** Arterial blood gas interpretation, ABG analysis, rules for rapid ABG analysis, Anion gap, Approach to mixed disorders

## Abstract

Disorders of acid–base balance can lead to severe complications in many disease states, and occasionally the abnormality may be so severe as to become a life-threatening risk factor. The process of analysis and monitoring of arterial blood gas (ABG) is an essential part of diagnosing and managing the oxygenation status and acid–base balance of the high-risk patients, as well as in the care of critically ill patients in the Intensive Care Unit. Since both areas manifest sudden and life-threatening changes in all the systems concerned, a thorough understanding of acid–base balance is mandatory for any physician, and the anesthesiologist is no exception. However, the understanding of ABGs and their interpretation can sometimes be very confusing and also an arduous task. Many methods do exist in literature to guide the interpretation of the ABGs. The discussion in this article does not include all those methods, such as analysis of base excess or Stewart’s strong ion difference, but a logical and systematic approach is presented to enable us to make a much easier interpretation through them. The proper application of the concepts of acid–base balance will help the healthcare provider not only to follow the progress of a patient, but also to evaluate the effectiveness of care being provided.

## Introduction

Arterial blood gas (ABG) analysis is an essential part of diagnosing and managing a patient’s oxygenation status and acid–base balance. The usefulness of this diagnostic tool is dependent on being able to correctly interpret the results. Disorders of acid–base balance can create complications in many disease states, and occasionally the abnormality may be so severe so as to become a life-threatening risk factor. A thorough understanding of acid–base balance is mandatory for any physician, and intensivist, and the anesthesiologist is no exception.

The three widely used approaches to acid–base physiology are the HCO_3_^-^ (in the context of pCO_2_), standard base excess (SBE), and strong ion difference (SID). It has been more than 20 years since the Stewart’s concept of SID was introduced, which is defined as the absolute difference between completely dissociated anions and cations. According to the principle of electrical neutrality, this difference is balanced by the weak acids and CO_2_. The SID is defined in terms of weak acids and CO_2_ subsequently has been re-designated as effective SID (SID_e_) which is identical to “buffer base.” Similarly, Stewart’s original term for total weak acid concentration (A_TOT_) is now defined as the dissociated (A^-^) plus undissociated (AH) weak acid forms. This is familiarly known as anion gap (AG), when normal concentration is actually caused by A^-^. Thus all the three methods yield virtually identical results when they are used to quantify acid–base status of a given blood sample.[1]

## Why is it Necessary to Order an ABG Analysis?

The utilization of an ABG analysis becomes necessary in view of the following advantages:

- Aids in establishing diagnosis.
- Guides treatment plan.
- Aids in ventilator management.
- Improvement in acid/base management; allows for optimal function of medications.
- Acid/base status may alter electrolyte levels critical to a patient’s status.

Accurate results for an ABG depend on the proper manner of collecting, handling, and analyzing the specimen. Clinically important errors may occur at any of the above steps, but ABG measurements are particularly vulnerable to preanalytic errors. The most common problems that are encountered include nonarterial samples, air bubbles in the sample, inadequate or excessive anticoagulant in the sample, and delayed analysis of a noncooled sample.

## Potential Preanalytical Errors

Preanalytical errors are caused at the following stages:

### During preparation prior to sampling

- Missing or wrong patient/sample identification;
- Use of the incorrect type or amount of anticoagulant
  - dilution due to use of liquid heparin;
  - insufficient amount of heparin;
  - binding of electrolytes to heparin;
- Inadequate stabilization of the respiratory condition of the patient; and
- Inadequate removal of flush solution in arterial lines prior to blood collection.

### During sampling/handling

- Mixture of venous and arterial blood during puncturing;
- Air bubbles in the sample. Any air bubble in the sample must be expelled as soon as possible after withdrawing the sample and before mixing with heparin or before any cooling of the sample has been done. An air bubble whose relative volume is up to 1% of the blood in the syringe is a potential source of significant error and may seriusly affect the pO_2_ value.
- Insufficient mixing with heparin.

### During storage/transport

- Incorrect storage
- Hemolysis of blood cells

#### General Storage Recommendation

- Do not cool the sample.[2]
- Analyze within 30 min. For samples with high *pa*O_2_ e.g., shunt or with high leukocyte or platelet count also analyze within 5 min.
- When analysis is expected to be delayed for more than 30 minutes, use of glass syringes and ice slurry is recommended.

### During preparation prior to sample transfer

- Visually inspect the sample for clots.
- Inadequate mixing of sample before analysis.

**Figure 1:**
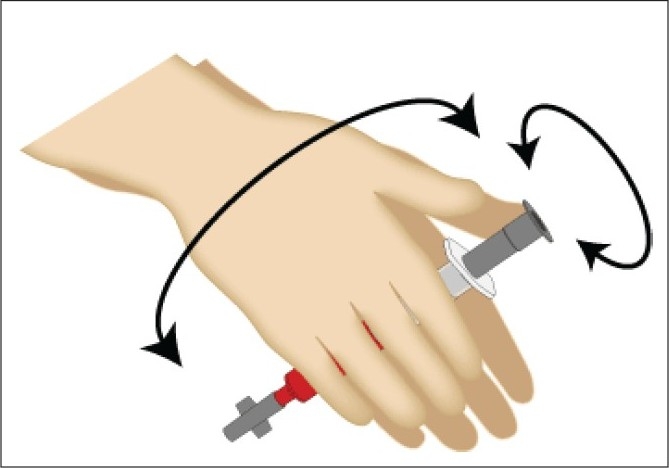
Correct method of mixing of the arterial sample with the anticoagulant in two dimensions to prevent stacking of red blood cells.

Insufficient mixing can cause coagulation of the sample. It is recommended to mix the blood sample thoroughly by inverting the syringe 10 times and rolling it between the palms as shown in Figure 1. This prevents stacking (such as coins or plates) of red blood cells.

### During anticoagulation

Modern blood gas syringes and capillary tubes are coated with various types of heparin to prevent coagulation in the sampler and inside the blood gas analyzer:

- Liquid nonbalanced heparin
- Dry nonbalanced heparin
- Dry electrolyte-balanced heparin (Na^+^, K^+^, Ca^2+^)
- Dry Ca^2+^-balanced heparin

Other anticoagulants, e.g., citrate and EDTA are both slightly acidic which increase the risk of pH being falsely lowered.

### Liquid heparin

The use of liquid heparin as the anticoagulant causes a dilution of the sample, i.e., dilutes the plasma, but not the contents of the blood cells. As a consequence, parameters such as *p*CO_2_ and electrolytes are affected. Only 0.05 mL of heparin is required to anticoagulate 1 mL of blood. Dead space volume of a standard 5 mL syringe with 1 inch 22 gauge needle is 0.2 mL; filling the syringe dead space with heparin provides sufficient volume to anticoagulate a 4-mL blood sample. If smaller sample volumes are obtained or more liquid heparin is left in the syringe, then the dilution effect will be even greater. The dilution effect also depends on the hematocrit value. Plasma electrolytes decrease linearly with the dilution of the plasma along with *p*CO_2_, cGlucose, and ctHb values. pH and *p*O_2_ values are relatively unaffected by dilution. *p*aO_2_ is only as little as 2% of the O_2_ physically dissolved in the plasma, and so the oximetry parameters given in fractions (or %) will remain unaffected.[3]

Syringes for blood gas analysis can have a wide range of heparin amounts.[4] The units are typically given as IU/mL (international units of heparin per milliliter) blood drawn into the syringe. In order to obtain a sufficient final concentration of heparin in the sample, blood volume recommended on the syringe must be drawn. Example: a syringe stated to contain 50 IU/mL when filled with 1.5 mL of blood means that the syringe contains a total 75 IU of dry heparin. If the user draws 2 mL of blood, then the resulting heparin concentration will be too low and the sample may coagulate. If the user draws only 1 mL, then the resulting heparin concentration will be higher than that aimed for, which may lead to producing falsely low electrolyte results.

Heparin binds to positive ions such as Ca^2+^, K^+^, and Na^+^. Electrolytes bound to heparin cannot be measured by ion-selective electrodes, and the final effect will be measurement offalsely low values. The binding effect and the resulting inaccuracy of results are especially significant for corrected Ca^2+^. The use of electrolyte-balanced heparin significantly reduces the binding effect and the resulting inaccuracy.[5]

The following steps for rapid interpretation of ABG are recommended:

#### Check for the consistency of ABG

While making an interpretation of an ABG always check for the consistency of the report by the modified Henderson equation.

$$\frac{\left[H^{+}-\right]\left[{\mathrm{HCO}}_{3}\right]}{{\mathrm{PaCO}}_{2}}\;=\;24$$

The hydrogen ion is calculated by subtracting the two digits after the decimal point of pH from 80, e.g., if the pH is 7.23 then

[H^+^] = 80 - 23 = 57

or

[H^+^] = 10^(9-pH)^

The hydrogen can be calculated from Table 1.

**Table 1:** pH value and corresponding H+ ion concentration

pH	H+	pH	H+
6.70	200	7.40	40
6.75	178	7.45	35
6.80	158	7.50	32
6.85	141	7.55	28
6.90	126	7.60	25
6.95	112	7.65	22
7.00	100	7.70	20
7.05	89	7.75	18
7.10	79	7.80	16
7.15	71	7.85	14
7.20	63	7.90	13
7.25	56	7.95	11
7.30	50	8.00	10
7.35	45		

#### Obtain a relevant clinical history

While making an interpretation of anABG, never comment on the ABG without obtaining a relevant clinical history of the patient, which gives a clue to the etiology of the given acid–base disorder. For example, a patient with a history of hypotension, renal failure, uncontrolled diabetic status, of treatment with drugs such as metformin is likely to have metabolic acidosis; a patient, with a history of diuretic use, bicarbonate administration, high-nasogastric aspirate, and vomiting, is likely to have metabolic alkalosis. Respiratory acidosis would occur in COPD, muscular weakness, postoperative cases, and opioid overdose, and respiratory alkalosis is likely to occur in sepsis, hepatic coma, and pregnancy.

#### Look at the oxygenation status of the patient

The oxygenation status of the patient is judged by the paO_2_;however, never comment on the oxygenation status without knowing the corresponding FiO_2_. Calculate the expected paO_2_ (generally five times the FiO_2_).[6]

Based on the expected paO_2_ classify as mild, moderate, and severe hypoxia.

#### Ventilatory status

Look at paCO_2_.

#### Acid–base status

Identify the primary disorder by looking at the pH

pH > 7.40–Alkalemia: – 7.40-Acidemia

Then look at paCO_2_ which is a respiratory acid, whether it is increased, i.e., >40 (acidosis) or decreased <40 (alkalosis) and if this explains the change of pH, then it is respiratory disorder; otherwise, see the trend of change of HCO_3_^-^(whether increased in alkalosis or decreased in acidosis)–if it explains the change of pH, then it is a metabolic disorder.

### In a normal ABG

- pH and paCO_2_ move in opposite directions.
- HCO_3_^-^and paCO_2_ move in same direction.

1. When the pH and paCO_2_ change in the same direction (which normally should not), the primary problem is metabolic; when pH and paCO_2_ move in opposite directions and paCO_2_ is normal, then the primary problem is respiratory.
2. Mixed Disorder–if HCO_3_^-^ and paCO_2_ change in opposite direction (which they normally should not), then it is a mixed disorder: pH may be normal with abnormal paCO_2_ or abnormal pH and normal paCO_2_).[7]

If the trend of change in paCO_2_ and HCO_3_^-^ is the same, check the percent difference. The one, with greater % difference, between the two is the one that is the dominant disorder.

e.g.: pH = 7.25 HCO_3_^-^=16 paCO_2_=60

Here, the pH is acidotic and both paCO_2_ and HCO_3_^-^ explain its acidosis: so look at the % difference

HCO_3_^-^% difference = (24 - 16)/24 = 0.33

paCO_2_% difference = (60 - 40)/40 = 0.5

Therefore, respiratory acidosis as the dominant disorder.

## Respiratory disorders

After the primary disorder is established as respiratory, then the following points will help us to approach further with regard to the respiratory disorder).[8]

- Ratio of rate of change in H^+^to change in paCO_2_
- Alveolar arterial oxygen gradient
- Compensation

### Ratio of rate of change in H^+^to change in paCO_2_

The above ratio of rate of change in H^+^to change in paCO_2_ helps in guiding us to conclude whether the respiratory disorder is acute, chronic, or acute on chronic. As we have seen, the hydrogen can be calculated from Table 1 and the change in H^+^ is calculated by subtracting the normal H^+^ from the calculated H^+^ ion.[9]

$$\frac{\Delta H^{+}}{\Delta {\mathrm{PaCO}}_{2}}<0.3–\mathrm{Chronic}$$

>0.8–acute

0.3–0.8–acute on chronic

### Alveolar Arterial Oxygen Gradient

It is calculated as follows:

$${\mathrm{PAO}}_{2}\;=\;{\mathrm{PiO}}_{2}\;-\;\frac{{\mathrm{PaCO}}_{2}}{R}$$

$${\mathrm{PiO}}_{2}={\mathrm{FiO}}_{2\;}\mathrm{(PB}-{\mathrm{PH}}_{2}\mathrm{O)}$$

$${\mathrm{PAO}}_{2}\;=\;{\mathrm{FiO}}_{2}\;\mathrm{(PB}-{\mathrm{PH}}_{2}\mathrm{O)}-\;\frac{{\mathrm{PaCO}}_{2}}{R}$$

where PAO_2_, alveolar partial pressure of oxygen; PiO_2_, partial pressure of inspired oxygen; FiO_2_, fraction of inspired oxygen; PB, barometric pressure (760 mmHg at sea level); PH_2_O, water vapor pressure (47 mm Hg), PaCO_2_, partial pressure of carbon dioxide in blood; R, respiratory quotient assumed to be 0.8.

$$=\;{\mathrm{FiO}}_{2}\;(760\;-\;47)\;-\;\frac{{\mathrm{PaCO}}_{2}}{0.8}$$

**Figure 2:**
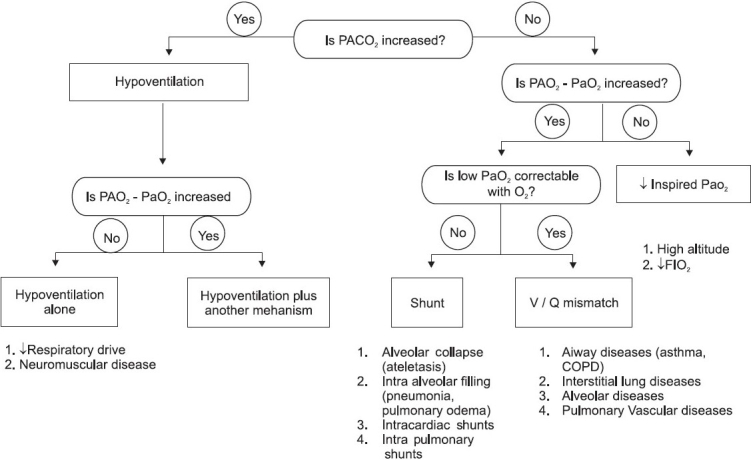
Flow diagram showing approach to hypoxemic respiratory failure

Hypoxemic respiratory failure can be associated with normal (10–15 mmHg) or increasedalveolar arterial oxygen gradient. Figure 2 shows the alogrithim for approach in a patient with hypoxemic respiratory failure. If this gradient is <20, then it indicates an extrapulmonary cause of respiratory failure.

Differentials of extrapulmonary causes of respiratory failure:

- Central nervous system–Respiratory center depression due to causes such as drug overdose, primary alveolar hypoventilation, and myxedema.
- Peripheral nervous system–Spinal cord diseases, Guillain-Barré syndrome, Amyotrophic lateral sclerosis.
- Respiratory muscles–Hypophosphatemia, muscle fatigue, myasthenia gravis, and polymyositis.
- Chest wall diseases–Ankylosing spondylitis, flail chest, thoracoplasty.
- Pleural diseases–Restrictive pleuritis
- Upper air way obstruction–Tracheal Stenosis, vocal cord tumor

### Compensation

#### Rules of compensation

1. The compensatory response depends upon the proper functioning of the organ system involved in the response (lungs or kidneys) and on the severity of acid–base disturbance. For example, the likelihood of complete compensation in chronic respiratory acidosis is <15% when paCO_2_ exceeds 60 mmHg.
2. Acute compensation occurs within 6–24 h and chronic within 1–4 days. Respiratory compensation occurs faster than metabolic compensation.
3. In clinical practice, it is rare to see complete compensation. The maximum compensatory response in most cases is associated with only 50–75% return of pH to normal. However, in chronic respiratory alkalosis, the pH may actually completely return to normalcy in some cases.

##### Respiratory acidosis

*Acute*: [HCO_3_^-^] increase by 1 mEq/L for every 10 mmHg increase in paCO_2_ above 40.

*Chronic*: [HCO_3_^-^] increase by 3.5 mEq/L for every 10 mmHg increase in paCO_2_ above 40.

##### Respiratory alkalosis

*Acute*: [HCO_3_^-^] decrease by 2 mEq/L for every 10 mmHg decrease in paCO_2_ below 40.

*Chronic*: [HCO_3_^-^] decrease by 5 mEq/L for every 10 mmHg decrease in paCO_2_ below 40.

## Metabolic disorders

In patients with metabolic acidosis, an excess of acid or loss of base is present. This causes the HCO_3_^-^:H_2_CO_3_ratio and pH to fall while no change occurs in pCO_2_–uncompensated metabolic acidosis.

As a result of compensatory mechanisms, the lungs in the form of CO_2_ excrete H_2_CO_3_ and the kidneys retain HCO_3_^-^. pCO_2_ falls and HCO_3_^-^: H_2_CO_3_ ratio and pH rise toward normal even though concentrations of HCO_3_^-^and H_2_CO_3_ are less than normal. This is called compensated metabolic acidosis and the expected paCO_2_ is calculated as paCO_2_ = [1.5 × HCO_3_+ 8] ± 2.

### Anion gap

For more than 40 years, the AG theory has been used by clinicians to exploit the concept of electroneutrality and has evolved as a major tool for evaluating the acid–base disorder. Anion gap is the difference between the charges of plasma anions and cations, calculated from the difference between the routinely measured concentration of the serum cations (Na^+^ and K^+^) and anions (Cl^-^ and HCO_3_^-^). Because electroneutrality must be maintained, the difference reflects the unmeasured ions. Normally, this difference or the gap is filled by the weak acids (A^-^) principally albumin, and to a lesser extent phosphates, sulfates, and lactates.

When the AG is greater than that produced by the albumin and phosphate, other anions (e.g., lactates and ketones) must be present in higher than normal concentration.

Anion gap = (Na^+^ + K^+^) - [Cl^-^ + HCO_3_^-^]

**Figure 3:**
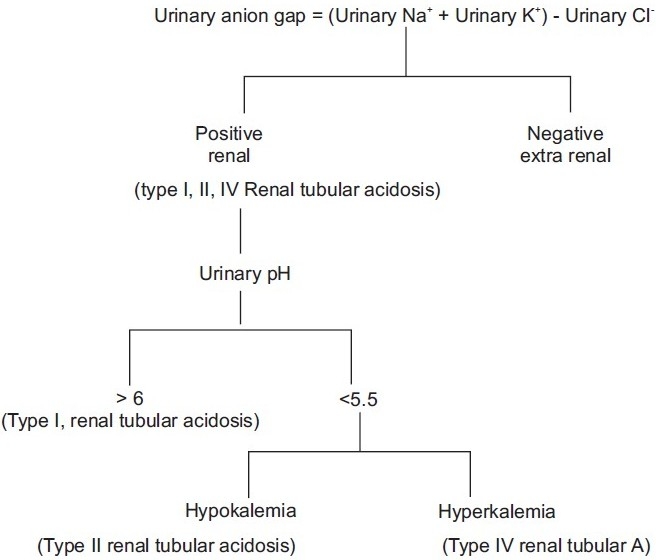
Approach to a patient with normal anion gap acidosis

Because of its low and narrow extracellular concentration, K^+^ is often omitted from the calculation The normal value ranges from 12 ± 4 when K^+^ is considered, and 8 ± 4 when K^+^ is omitted. Figure 3 shows the alogrithm for the approach to patients with normal AG acidosis.

The primary problem with AG is its reliance on the use of the normal range produced by the albumin and to a lesser extent phosphate, the level of which may be grossly abnormal in critically ill patients. Because these anions are not strong anions, their charges will be altered by changes in pH.[10,11]

#### Serum protein and phosphate

Normal AG = 2{albumin(gm/L)} + 0.5 {phosphate (mg/dL)}

Acid–base status

In Acidemic state - Anion gap decreases by 1-3

In Alkalemic state - Anion gap increases by 2-5

#### Major clinical uses of the anion gap

- For signaling, the presence of a metabolic acidosis and confirm other findings.
- Helping to differentiate between causes of metabolic acidosis: High AG versus normal AG metabolic acidosis. In an inorganic metabolic acidosis (e.g., due to HCl infusion), the infused Cl^-^ replaces HCO_3_^-^, and the AG remains normal. In an organic acidosis, the lost bicarbonate is replaced by the acid anion which is not normally measured. This means that the AG is increased.
- Providing assistance in assessing the biochemical severity of the acidosis and follow the response to treatment.

Disorders that are associated with a low or negative serum AG are listed in Table 2.

**Table 2:** Disorders associated with low serum anion gap

Cause	Comments
Laboratory error	Most frequent cause of low anion gap
Hypoalbuminemia	Second most common cause of low serum anion gap
Multiple myeloma	Level of anion gap correlates with serum concentration of paraprotein
Halide intoxication	Anion gap depends on serum halide concentration
(bromide, lithium, iodide)	(low anion gap with lithium ≥4 mEq/L)
Hypercalcemia	more likely in hypercalcemia associated with 10 hyperparathyroidism
Hypermagnesemia	Theoretical cause but not documented in literature
Polymyxin B	Anion gap depends on serum level; occurs with preparation with chloride
Underestimation of serum sodium	Most frequent with hypernatremia or hypertriglyceridemia
Overestimation of serum chloride	Rare with ion selective electrodes
Overestimation of serum bicarbonate	Spurious ⁭in serum HCO_3_ if cells not separated from sera

Table 3 elaborates the species of the unaccounted anions along with their sources of origin and diagnostic adjunts in case of high AG metabolic acidosis.

**Table 3:** Description of the species of unmeasured anions, source of origin, and diagnostic adjuncts in case of high anion gap metabolic acidosis

Cause	High serum anion gap		
	Comments		
	Species	Origin	Diagnostic adjuncts
Renal failure	Phosphates, sulphates	Protein metabolism	BUN/creatinine
Ketocidosis	Ketoacids	Fatty acid metabolism	Serum/urine ketones
Diabetic	β Hydroxybutyrate		
Alcoholic		
Starvation	Acetoacetate		
Lactic acidosis	Lactate		Lactate levels
Exogenous poisoning	Salicylate	Salicylate	Concomitant
	Lactate	Respiratory and metabolic alkalosis	
	ketoacids		

In the patients with metabolic alkalosis, there is an excess of base or a loss of acid which causes the HCO_3_^-^:H_2_CO_3_ ratio and pH to rise, but with no change occurring in pCO_2_, which is called uncompensated metabolic alkalosis. However, the kidney has a large capacity to excrete excess bicarbonate and so, for sustaining the metabolic alkalosis, the elevated HCO_3_^-^concentration must be maintained through an abnormal renal retention of HCO_3_^-^.

Compensatory respiratory acidosis may be so marked that pCO_2_ may rise higher than 55 mmHg. Expected paCO_2_is calculated as paCO_2_ = [0.7 × HCO_3_^-^+ 21] ± 2 or 40 + [0.7 ΔHCO_3_]. This is called compensated metabolic alkalosis.

Most of the patients with metabolic alkalosis can be treated with chloride ions in the form of NaCl (saline responsive) rather than KCl (which is preferable). When NaCl is given, Cl^-^ions are supplied, and so the blood volume increases and the secretion of aldosterone in excess decreases. Thus, excessive urinary loss of K^+^and excessive reabsorption of HCO_3_^-^ stops. When metabolic alkalosis is due to the effects of excessive aldosterone or other mineralocorticoids, the patient does not respond to NaCl (saline resistant) and requires KCl.

Based on the urinary chloride, metabolic alkalosis is divided into:

Chloride responsive or extracellular volume depletion (urinary chloride < 20)

- Vomiting
- Diuretic
- Post hypercapnic
- Chronic diarrhea
- Chloride resistant (urinary chloride > 20)
- Severe potassium depletion
- Mineralocorticoid excess–Primary hypealdosteronism, Cushing’s Syndrome, Ectopic ACTH
- Secondary hypereldosteronism–Renovascular disease, malignant hypertension, CHF, cirrhosis

#### Aproach to mixed disorder

Mixed metabolic disturbances (e.g., high AG from diabetic ketoacidosis plus normal AG from diarrhea) can be identified using the relationship between AG and HCO_3_^-^, which is called the gap–gap ratio. It is the ratio of change in anion gap (ΔAG) to change in HCO_3_^-^ (ΔHCO_3_^-^). When hydrogen ions accumulate in blood, the decrease in serum HCO_3_^-^ is equivalent to the increase in AG and the increase in AG excess/HCO_3_^-^ deficit ratio is unity, i.e., pure increase in AG metabolic acidosis. When a normal AG acidosis is present, the ratio approaches zero. When a mixed acidosis is present (high AG + normal AG), the gap–gap ratio indicates the relative contribution of each type to the acidosis. If it is <1, then it suggests that there is a normal AG metabolic acidosis associated with it and if >2 it suggests that there is associated metabolic acidosis.

#### Rules for rapid clinical interpretation of ABG

When required to make a proper approach towards the evaluation of blood gas and acid–base disturbances in the body, the following scheme is suggested:

1. Look at pH - < 7.40 - Acidosis; > 7.40 - Alkalosis
2. If pH indicates acidosis, then look at paCO_2_and HCO_3_^-^
3. If paCO_2_is ↑, then it is primary respiratory acidosis
  - To determine whether it is acute or chronic
    ΔH^+^ / ΔpaCO_2_ <0.3–chronic
    >0.8–acute
    0.3-0.8–acute on chronic
  - Calculate compensation by the respective methods
    *Acute*: [HCO_3_^-^] ↑ by 1 mEq/L for every 10 mmHg ↑ in paCO2 above 40.
    *Chronic*: [HCO_3_^-^] ↑ by 3.5 mEq/L for every 10 mmHg ↑ in paCO2 above 4
4. If paCO_2_↓ and HCO_3_^-^ is also ↓→ primary metabolic acidosis
  Calculate expected paCO_2_as follows:
  paCO_2_ = [1.5 × HCO_3_+ 8] ± 2 metabolic acidosis only
  paCO_2_ < expected paCO_2_→ concomitant respiratory alkalosis.
  paCO_2_ > expected paCO_2_→ concomitant respiratory acidosis
5. If HCO_3_^-^is ↓, then AG should be examined.
6. If AG is unchanged → then it is hyperchloremic metabolic acidosis.
7. If AG is ↑ → then it is wide AG acidosis.
8. Check gap-gap ratio
  ΔAG/Δ HCO3^-^ = 1, pure increased AG metabolic acidosis
  <1 normal anion gap metabolic acidosis
  >2 associated metabolic acidosis.
9. If pH indicates alkalosis, then look at HCO_3_^-^ and paCO_2_.
10. If paCO_2_is ↓ → then it is primary respiratory alkalosis.
  - Whether it is acute or chronic (with the same formula as above)
  - Calculate compensation by the respective methods:
    *Acute*: [HCO_3_^-^]↓ by 2 mEq/L for every 10 mmHg
    ↓ in paCO_2_below 40.
    *Chronic*: [HCO_3_^-^] ↓ by 5 mEq/L for every
    10mmHg ↓ in paCO_2_ below 40.
11. If paCO_2_ ↑ and HCO_3_^-^ also ↑ → then it is primary metabolic alkalosis.
  Calculate the expected paCO_2_
  paCO_2_ = [0.7 × HCO3^-^+ 21] ± 2 Or 40 + [0.7 ΔHCO3] → metabolic alkalosis only
  paCO_2_ < expected paCO2 → concomitant respiratory alkalosis.
  paCO_2_ > expected paCO2 → concomitant respiratory acidosis
12. Check urinary chloride
  if urinary chloride < 20 → chloride responsive or ECV depletion
  if urinary chloride > 20→ chloride resistant
13. If pH is normal ABG may be normal or mixed disorder
  - ↑paCO_2_ and ↓HCO_3_^-^→ respiratory and metabolic acidosis
  - (b) ↓paCO_2_ and↑ HCO_3_^-^→ respiratory and metabolic alkalosis.
    Calculate % difference (ΔHCO_3_^-^/HCO_3_^-^and ΔpaCO_2_/paCO_2_) to see which is dominant disorder.
